# Establishing a Pediatric Acute-Onset Neuropsychiatric Syndrome Clinic: Baseline Clinical Features of the Pediatric Acute-Onset Neuropsychiatric Syndrome Cohort at Karolinska Institutet

**DOI:** 10.1089/cap.2018.0127

**Published:** 2019-10-07

**Authors:** Caroline Gromark, Robert A. Harris, Ronny Wickström, AnnaCarin Horne, Maria Silverberg-Mörse, Eva Serlachius, David Mataix-Cols

**Affiliations:** ^1^ Department of Clinical Neuroscience, Karolinska Institutet, Stockholm, Sweden.; ^2^ BUP OCD and Related Disorders, Stockholm Health Care Services, Stockholm County Council, Stockholm, Sweden.; ^3^ Centre for Psychiatry Research, Stockholm Health Care Services, Stockholm County Council, Stockholm, Sweden.; ^4^ Department of Women's and Children's Health, Karolinska Institutet, Stockholm, Sweden.; ^5^ Division of Pediatric Neurology, Karolinska University Hospital, Solna, Sweden.; ^6^ Division of Pediatric Rheumatology, Karolinska University Hospital, Solna, Sweden.

**Keywords:** PANS, PANDAS, OCD, tics, autoimmune disease

## Abstract

***Objectives:*** Pediatric acute-onset neuropsychiatric syndrome (PANS) is a descriptive clinical entity defined by the abrupt onset of psychiatric and somatic symptoms leading to significant loss of function. Data on well-characterized PANS patients are limited, biomarkers have yet to be identified, and a solid evidence base to guide treatment is lacking. In this study, we present our experience of a systematic evaluation of the first 45 patients included in a Swedish cohort.

***Methods:*** During the period 2014–2018, our clinic received 100 referrals regarding suspected PANS. All patients underwent a standardized psychiatric/medical evaluation by a child/adolescent psychiatrist and a clinical psychologist or a nurse. Those with severe symptoms were also assessed by a pediatric neurologist and a pediatric rheumatologist. Laboratory tests were obtained at different time points in an attempt to capture an active disease state.

***Results:*** Of the 100 referrals, 45 met strict PANS criteria and consented to participate in a long-term follow-up study. The median age at intake was 7.2 years (range 3.0–13.1) and 56% were male. Ninety-three percent fulfilled both criteria for acute/atypical onset of PANS symptoms and having had an infection in relation to onset. Sixteen percent had an onset of an autoimmune or inflammatory disorder in temporal relation to the onset of PANS-related symptoms. The most common onset symptoms were obsessive-compulsive disorder (89%), anxiety (78%), and emotional lability (71%). Twenty-four percent had a preexisting autoimmune disease (AD) and 18% a preexisting psychiatric/neuropsychiatric diagnosis. Sixty-four percent of biological relatives had at least one psychiatric disorder and 76% at least one AD or inflammatory disorder. Complement activation (37%), leukopenia (20%), positive antinuclear antibodies (17%), and elevated thyroid antibodies (11%) were the most common laboratory findings.

***Conclusions:*** In our PANS cohort, there was a strong indication of an association with AD. Further work is needed to establish whether any of the potential biomarkers identified will be clinically useful. Long-term follow-up of these patients using the Swedish national registers will enable a deeper understanding of the course of this patient group.

## Introduction

There is increasing evidence for an association between autoimmune disease (AD) and neuropsychiatric disorders (Najjar et al. 2013; Mataix-Cols et al. 2018). Pediatric acute-onset neuropsychiatric syndrome (PANS) is a descriptive entity of disputed validity and for which there are currently no defined biomarkers (Chang et al. 2015; Hesselmark and Bejerot 2017a, 2017b). It affects young children, often in temporal relation to an uncomplicated infection, resulting in abrupt onset of obsessive-compulsive disorder (OCD) and/or anorexia, emotional lability, and a wide range of somatic symptoms (Swedo et al. 1998; Chang et al. 2015; Hesselmark and Bejerot 2017a, 2017b). The severity of symptoms may lead to significant loss of function, impacting both the children themselves and their families (Frankovich et al. 2015). Other diseases of known etiology, but with a similar clinical picture, such as Sydenham's chorea, systemic lupus erythematosus, or other inflammatory encephalitides, such as anti-NMDA receptor encephalitis, need to be excluded (Dalmau et al. 2008; Dale and Brilot 2012; Hacohen et al. 2013; Ramanathan 2014). While the etiology is unknown, infectious agents such as group A streptococci (GAS), mycoplasma, and Epstein–Barr virus as well as AD and inflammatory disorders have been described as contributing to the pathogenic mechanisms and potential triggers for the constellation of symptoms that constitute PANS (Kurlan et al. 2008; Leckman et al. 2011; Brimberg et al. 2012; Cutforth et al. 2016; Mahony et al. 2017a, 2017b).

Based on clinical experience and previous research, the PANS Research Consortium (Cooperstock et al. 2017; Frankovich et al. 2017; Thienemann et al. 2017) has developed expert consensus guidelines for psychiatric, infectious, and immunomodulatory treatments, respectively, but the lack of well-defined cohorts of patients, absence of reliable biomarkers, and lack of conclusive clinical trials have complicated the interpretation of results. Clinical data of well-characterized PANS patients are still limited and more evidence is needed to support the current treatment routines that have been developed mainly according to clinical experience, case reports, and case series. Previous research has suggested that immunomodulatory and/or anti-inflammatory treatments may be beneficial in some cases, but evidence is inconclusive (Latimer et al. 2015; Farhood et al. 2016; Williams et al. 2016; Brown et al. 2017a, 2017b; Spartz et al. 2017; Sigra et al. 2018). Definitive clinical trials are sorely needed to guide clinical decision-making.

Our specialist OCD and related disorders clinic started accepting potential PANS referrals in November 2014, and since then, we have gathered a well-defined cohort of patients fulfilling criteria for PANS, together with a large amount of longitudinal clinical and biological data. We herein describe development of the PANS clinic and preliminary clinical and laboratory data for the first 45 patients included in the cohort. This research is part of a larger project that will gather long-term follow-up data from these patients through linkage with the Swedish population-based registers, with the aim of identifying potential clinical and biological predictors of clinical course for this group.

## Methods

### Clinical setting

All study participants were recruited from a specialist pediatric OCD and related disorders outpatient clinic in Stockholm, Sweden. The clinic primarily receives referrals from Child and Adolescent Psychiatry Services (CAMHS) and pediatric services across the entire Stockholm region and occasionally from other Swedish regions and Nordic countries. All patients and their parents/legal guardians routinely fill in questionnaires before their first appointment with the multidisciplinary clinical team comprising child/adolescent psychiatrists, clinical psychologists, and nurses. This information is then used to conduct a more focused and efficient, face-to-face diagnostic assessment. During the first 3-hour appointment, detailed sociodemographic and clinical information is gathered from patients and their parents and clinical diagnoses are made according to *International Classification of Diseases, 10th Revision* (ICD-10), and *Diagnostic and Statistical Manual of Mental Disorders, 5th edition* (DSM-5), criteria (World Health Organization 2011; American Psychiatric Association 2013). After this assessment, patients are either offered treatment at the clinic or referred to more appropriate services. For all patients undertaking treatment at the clinic, assessments are repeated at post-treatment and at several fixed follow-up times: 3, 6, and 12 months after the end of the treatment. All patients assessed at the clinic are routinely asked to participate in research studies, including a long-term follow-up project with aims to evaluate the broad long-term outcomes of our patients with the help of the Swedish population-based registers.

In 2014, the clinic started accepting referrals of potential PANS cases and, as the demand increased, we established a PANS team within our clinic, currently consisting of a child and adolescent psychiatrist, a nurse, and two clinical psychologists. The PANS team closely collaborates with the pediatric neuroinflammation team at the Karolinska University Hospital, which creates a multispecialist environment with child and adolescent psychiatry, pediatric rheumatology, and pediatric neurology. The collaboration has enabled development of Sweden's first clinical routines for evaluation and management of youths with PANS in consensus with pediatric neurology, pediatric rheumatology, and CAMHS across Stockholm in April 2018. These clinical routines resemble, but are not identical to, other guidelines recently reported in the United States (Cooperstock et al. 2017; Frankovich et al. 2017; Thienemann et al. 2017). Verified infections are treated with antibiotics, but because clinical trials are still inconclusive regarding the benefits of long-term antibiotics, the Stockholm clinical routines discourage their prophylactic use until firmer evidence becomes available. The treatment routines also include a requirement for neurological clinical signs—electroencephalography (EEG) and/or magnetic resonance imaging (MRI) abnormalities and/or biomarkers (in blood and/or cerebrospinal fluid [CSF])—that suggest an active neuroinflammation before intravenous immunoglobulin (IVIG) treatment is considered.

All young people and their parents gave written consent to participate in the current study, which was approved by the Regional Ethics Review Board in Stockholm (reference number EPN 2015/1977-31/4).

### Clinical evaluations

All suspected PANS cases underwent a thorough psychiatric and medical evaluation at first presentation at the clinic. A child and adolescent psychiatrist, a clinical psychologist, and a specialist psychiatric nurse carried out the assessments. The psychiatric evaluation included a full developmental and psychiatric history as well as relevant validated rating scales depending on primary symptoms (such as the Children's Yale–Brown Obsessive Compulsive Scale [CYBOCS] for OCD or the Yale Global Tic Severity Scale [YGTSS] for tics) (Goodman et al. 1989; Leckman et al. 1989; Storch et al. 2010; McGuire et al. 2018). A clinician assessed global psychiatric symptom severity and improvement at each visit with the Clinical Global Impressions-Severity Scale (CGI-S) and the Clinical Global Impressions-Improvement Scale (CGI-I), respectively. In this study, CGI-S and CGI-I were employed as measures of general psychopathology, rather than measures of specific forms of psychopathology. Global functioning was assessed at each visit using the Children's Global Assessment Scale (CGAS) (Shaffer et al. 1983; Busner and Targum 2007). As the study progressed, it became apparent that time-consuming instruments such as the CYBOCS and YGTSS were difficult to administer to our youngest patients (as young as 3 or 4 in our cohort), some of whom had difficulties communicating their symptoms. Parents are often unaware of the child's obsessions and can only report on observable behavior. Finally, PANS patients often present with a wide range of complaints, sometimes OCD being the least prominent. For these reasons, we chose not to use the CYBOCS as part of our routine baseline assessment, unless OCD was the main clinical presentation and the patient was old and/or communicative enough to be accurately assessed.

The medical evaluation included developmental and medical history, vaccination records, a thorough somatic assessment focusing on signs of infection, and rheumatological and neurological signs. Depending on severity of symptoms and clinical signs, further assessments such as EEG, MRI, or lumbar puncture for CSF were arranged through pediatric neurology and/or rheumatology clinics. Family history was carefully recorded in a systematic way using a comprehensive checklist of psychiatric, neurological, and autoimmune/inflammatory disorders in first-, second-, and third-degree relatives.

Neuropsychological testing was performed if the patient showed signs of cognitive impairment, if the disease had a deteriorating course, and/or to evaluate treatment outcomes. Medical records, including those from primary care physicians, emergency care visits, and inpatient units, were reviewed by the treating clinician responsible for the PANS team (C.G.).

### Laboratory workup

The team developed a laboratory protocol that was used for every patient. The protocol included basic blood measurements of C-reactive protein (CRP), erythrocyte sedimentation rate, hemoglobin, and complete blood count (CBC), indicators for liver and kidney function, thyroid tests, celiac test, and rheumatological markers such as antinuclear antibodies (ANAs), histone antibodies, protein fractions, immunoglobulins, complements, and serum amyloid A (SAA), as well as some of the more frequently tested cytokines (interleukin [IL]-1-β, IL-6, IL-8, and tumor necrosis factor-α [TNF-α]). The reference values used were those used clinically by the Karolinska laboratory (Bäck et al. 1999, Roche Diagnostics GmbH 2009, Yamada 1999). Blood tests were ordered at the time of first assessment at the clinic and at some intervals during follow-up, more frequently if clinically necessary because of a more severe disease trajectory. When the patient exhibited signs of infection, cultures were taken. The laboratory workup evolved slightly over the course of the study period, following the clinical development of PANS care. Some tests, such as measurement of histone antibodies, cytokines, and SAA, have therefore not been conducted for all patients.

The laboratory results reported in this article represent findings at one point in time during onset, flare (defined as CGI-S equal to or higher than 4 and marked as a worsening period in patient records), or during a chronic-static or progressive course. If the full laboratory workup was made repeatedly for the same patient, the point with the highest CGI-S was chosen. In the few cases where CGI-S was not sufficient to differentiate the severity of flares, the full symptom description of the flare as described in patient records was assessed. We therefore believe that the findings represent, as far as possible, an active disease state. Some patients showed a positive ANA, complement activation, and/or elevated SAA at another point in the disease course than the one chosen, for example, during a less severe flare. These findings have not been included in the table. Patients with diagnosed celiac disease, eating gluten-free and therefore having no detectable transglutaminase antibodies, have been included as being transglutaminase antibody negative if the laboratory test at the time point was negative. Streptococcal cultures were only included if taken at the same time point as the other laboratory tests.

### Statistical analyses

Statistical analysis was conducted using STATA software (version STATA/IC15.1 for Mac). Analyses were largely descriptive in nature. When comparing subgroups of patients within the cohort, t-tests for continuous variables and chi-square tests for proportions were employed. A *p*-value below 0.05 was considered statistically significant.

## Results

### Referral flow

**FIG. 1.**
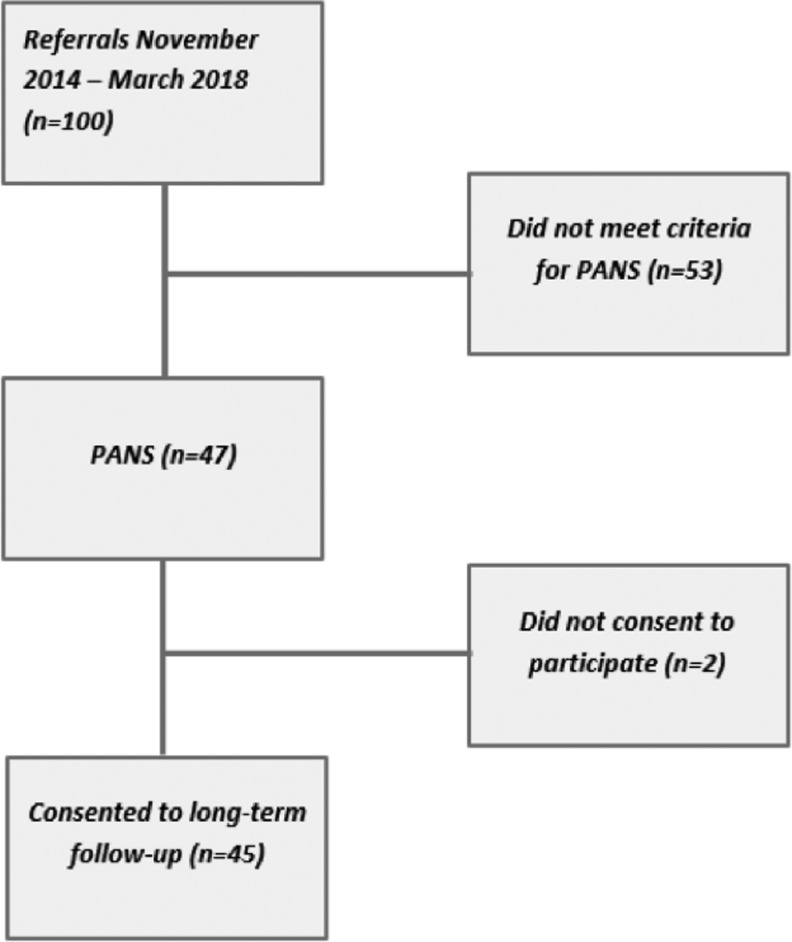
Flow chart of study participants. PANS*:* individuals fulfilling PANS criteria. Did not meet criteria for PANS*:* individuals not fulfilling PANS criteria due to absence of core symptoms such as obsessive-compulsive disorder and/or anorexia, insidious symptom onset, lack of somatic signs, or spontaneous resolution of symptoms before the assessment. PANS, pediatric acute-onset neuropsychiatric syndrome.

Between November 2014 and March 2018, our clinic received 100 referrals regarding suspected PANS. Forty-seven patients met the criteria for PANS, even though it was difficult to assess the acuteness of onset in some cases. Of these, 45 consented to participate in our long-term follow-up study and future linkage to the Swedish registers (Fig. 1). The most common reasons for exclusion were absence of core psychiatric symptoms such as OCD and/or anorexia, insidious symptom onset, absence of somatic signs, and/or spontaneous resolution of symptoms before the assessment took place.

### Demographic and clinical characteristics

The sociodemographic and clinical characteristics of the cohort are presented in Table 1. Fifty-six percent of patients were male. The mean age at symptom onset was 7.5 years (standard deviation [SD] 2.5, range 3.0–13.1, median 7.2 years) and the mean age at presentation at the clinic/first assessment was 9.0 years (SD 3.1, range 3.3–15.7, median 9.0 years). Developmental abnormalities were noted in patient records in 18% of the cohort, the same fraction as having had a preexisting psychiatric and/or neuropsychiatric diagnosis. A slightly higher fraction, 24%, had a preexisting disease or disorder commonly considered to have an autoimmune or inflammatory etiology, the most common being severe asthma, severe atopic eczema, severe and multiple nutritional allergies, celiac disease, postinfectious arthritis, and Henoch-Schönlein's purpura.

**Table 1.** Patient Characteristics at Intake (*n* = 45)

*Patient demographics*	*Frequencies/means (SD)*
Male	25/45 (56%)
Mean age at symptom onset (years)	7.5 (SD 2.5)
Mean age at intake (years)	9.0 (SD 3.1)
Developmental abnormalities (psychomotor, language disorder, and/or learning disability)	8/45 (18%)
Preexisting psychiatric/neuropsychiatric diagnoses	8/45 (18%)
Preexisting autoimmune disease or inflammatory disorder	11/45 (24%)
CGAS at intake^a^	50 (SD 10.1)
CGI-S at intake	3.8 (SD 0.9)
Acute symptom onset	42/45 (93%)
Infection in temporal relation to symptom onset	42/45 (93%)
Onset of autoimmune disease or inflammatory disorder in temporal relation to symptom onset	7/45 (16%)

^a^ Available for 43 patients only.

CGAS, Children's Global Assessment Scale; CGI-S, Clinical Global Impressions-Severity Scale; SD, standard deviation.

Almost all patients (93%) clearly fulfilled both criteria for acute onset (<72 hours) of PANS-related symptoms and having had an infection in temporal relation to symptom onset. For the remaining 7% of cases, it was not possible to fully confirm the type of onset because of a long time having passed between onset and presentation at the clinic or insufficiently detailed patient records, but the parent-reported clinical history was considered distinct enough for the patient to be included in the cohort. Sixteen percent had an onset of an autoimmune or inflammatory disorder in temporal relation to the onset of PANS-related symptoms. Among these disorders, autoimmune thyroiditis, celiac disease, and diabetes mellitus type 1 were represented.

### Onset symptoms

**FIG. 2.**
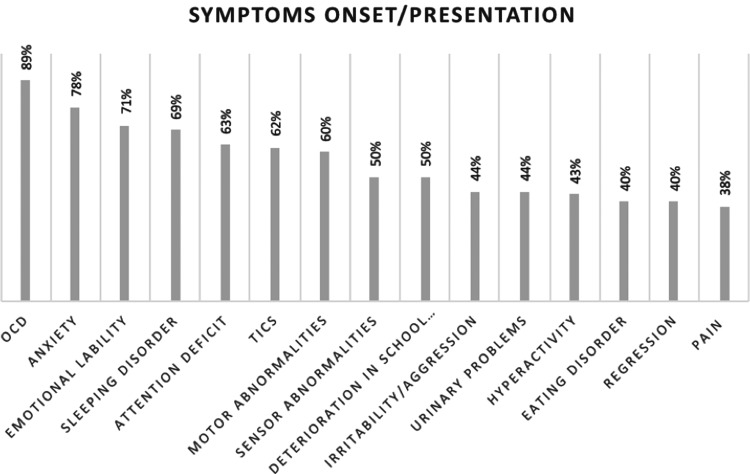
Onset symptoms as described in patient records and/or at presentation at the clinic (*n* = 45). Values do not add up to 100% since most patients had more than one symptom at onset. OCD, obsessive-compulsive disorder.

The main onset symptoms are presented in Figure 2. In the cohort, 89% were assessed to fulfill criteria for OCD at the time of onset. Anxiety (including separation anxiety) was evident in 78% and emotional lability and/or depression in 71%. Sleep disorders, including insomnia, sleep disturbances, and frequent nightmares, were reported by 69% of patients. Sixty-two percent had complex tics, which were often both motor and vocal, resulting in pain or loss of function. Other forms of motor abnormalities (including chorea and choreiform movements, dystonia, perception of muscle weakness, and difficulties with gross motor control as well as fine motor skills) were present in 60% of patients. Attention deficit was reported by 63% and hyperactivity by 43% of patients. Despite the young age of the cohort, 50% reported deterioration in school performance since symptom onset. Irritability/aggression and regressive behaviors were reported in 44% and 40%, respectively. Half of the patients reported sensory abnormalities, including hypersensitivity to touch/clothes, sound, and light. Forty percent had an eating disorder (significant loss of appetite resulting in weight loss, avoidant–restrictive food intake disorder, or other OCD-related eating disorder). Somatic symptoms such as urinary problems and pain were reported in 44% and 38%, respectively.

The mean CGAS and CGI-S values at presentation were 50 (SD 10.1, range 21–70) and 3.8 (SD 0.9, range 2–6), respectively. The wide range in CGAS rating indicates that some patients were severely ill at onset, while others were mildly ill, but still fulfilled PANS criteria (Table 1).

### Somatic signs

Somatic signs at onset/presentation are presented in Table 2. Slightly fewer than half of the patients, 46%, exhibited ear, nose, and throat (ENT) infection signs at onset/first assessment. Nine percent had palatal petechiae as a suspected sign of streptococcal infection (Mahony et al. 2017b). Skin abnormalities, including dermatographia, livedo, rashes, and/or eczema, were present in 61% of patients. From the somatic assessment, 23% had some kind of neurological abnormality (the most common being ataxia or side differences in force or reflexes of the extremities), 23% had motor abnormalities, and 23% exhibited choreiform movements. Choreatic movements at the time of first assessment were only evident in one patient (1/41; 2%). Only one patient (1/28; 4%) presented with a heart abnormality upon auscultation. It consisted of a heart murmur, which after ultrasound assessment, was considered physiological and not due to inflammatory changes.

**Table 2.** Somatic Findings at First Clinical Evaluation

*Somatic sign onset/presentation*	*Frequencies*^a^
Heart abnormalities	1/28 (4%)
Lung abnormalities	0/27 (0%)
Stomach abnormalities	0/13 (0%)
Joint abnormalities	0/16 (0%)
ENT infection signs	16/35 (46%)
Palatal petechiae	2/22 (9%)
Skin abnormalities (dermatographia, livedo, severe dryness, redness, rash, and eczema, etc.)	11/18 (61%)
Neurological abnormalities	7/31 (23%)
Abnormalities in motor function	7/30 (23%)
Chorea	1/41 (2%)
Choreiform movements	6/26 (23%)

^a^ These signs were only assessed in a subsample of participants.

ENT, ear, nose, and throat.

### Laboratory findings

Laboratory findings are presented in Table 3. The most common CBC abnormality was mild leukopenia (9/45; 20%). Ferritin deficiency and vitamin D deficiency were frequently noted in the cohort, but are both relatively frequent in the normal pediatric population (Parkin et al. 2017; Yesiltepe and Hatun 2018). In the total cohort, 11% had elevated thyroperoxidase antibodies (anti-TPO) and 5% had elevated transglutaminase antibodies, indicating thyroid and celiac disease, respectively. A full 37% had complement deficiencies indicating complement activation. Seventeen percent had positive ANAs and 14% had an elevated SAA. Eleven percent had low immunoglobulin G (IgG) and 9% had low immunoglobulin A (IgA). None of the patients had low immunoglobulin M (IgM). None of the patients had a clear increase in any of the serum cytokines tested for (IL-1-β, IL-6, IL-8, and TNF-α). Streptococcal cultures were rarely taken at the same time as the blood tests, but when performed were positive for GAS in 50% of the 10 tested cases.

**Table 3.** Laboratory Findings Corresponding to One Point in Time During Symptom Onset, Flare (Defined as Clinical Global Impressions-Severity Scale Equal to or Higher Than 4 and Marked as a Worsening Period in Patient Records), or During a Chronic-Static or Progressive Course, Compared with Population Norms

*Laboratory findings*	*Frequencies*^a^
CBC abnormalities	26/45 (58%)
Elevated ESR	6/43 (14%)
Elevated CRP	4/37 (11%)
Low ferritin	8/39 (21%)
Low vitamin D	10/36 (28%)
TSH abnormalities	4/40 (10%)
Low T4	0/40 (0%)
Anti-TPO	4/38 (11%)
Transglutaminase antibodies	2/40 (5%)
ANAs	7/41 (17%)
Histone antibodies	0/31 (0%)
Elevated SAA	3/22 (14%)
Complement activation	13/35 (37%)
Low IgG	4/36 (11%)
Low IgA	3/34 (9%)
Low IgM	0/34 (0%)
Elevated IL-1-β	0/25 (0%)
Elevated IL-6	1/25 (4%)
Elevated IL-8	0/25 (0%)
Elevated TNF-α	0/24 (0%)
Positive strep/throat culture	5/10 (50%)

^a^ These tests were only assessed in a subsample of participants.

ANAs, antinuclear antibodies; CBC, complete blood count; CRP, C-reactive protein; ESR, erythrocyte sedimentation rate; IgA, immunoglobulin A; IgG, immunoglobulin G; IgM, immunoglobulin M; IL, interleukin; SAA, serum amyloid A; TNF-α, tumor necrosis factor-α; TPO, thyroperoxidase; TSH, thyroid-stimulating hormone.

### Family history of psychiatric, autoimmune, and inflammatory disorders

Data of family history of psychiatric and AD/inflammatory disorders are presented in Table 4, which combines data for first-, second-, and third-degree relatives. In all the relatives combined, 64% had at least one psychiatric disorder, the most common being depression (36%), attention-deficit/hyperactivity disorder (20%), OCD (16%), anxiety (16%), tics (13%), and autism spectrum disorder (11%).

**Table 4.** Family History of Psychiatric Disorders and Autoimmune Diseases in First-, Second-, and Third-Degree Relatives of Study Participants (*n* = 45)

*Family history*	*Frequencies*
Psychiatric/neuropsychiatric disorder in first-, second-, and third-degree relatives	
Attention-deficit/hyperactivity disorder	9/45 (20%)
Autism spectrum disorder	5/45 (11%)
Tics	6/45 (13%)
Obsessive-compulsive disorder	7/45 (16%)
Anxiety disorders	7/45 (16%)
Depression	16/45 (36%)
Any	29/45 (64%)
Autoimmune disease/inflammatory disorder in first-, second-, and third-degree relatives	
Thyroid disease	12/45 (27%)
Celiac disease	2/45 (4%)
SLE	0/45 (0%)
Rheumatoid arthritis/juvenile idiopathic arthritis	11/45 (24%)
Psoriasis	7/45 (16%)
Diabetes mellitus type 1	2/45 (4%)
Inflammatory bowel disease	3/45 (7%)
Neuroinflammatory disease (multiple sclerosis and amyotrophic lateral sclerosis)	4/45 (9%)
Other (vitiligo, IgA nephritis, vasculitis, polymyalgia rheumatica, or rheumatic fever)	20/45 (44%)
Any	34/45 (76%)

SLE, systemic lupus erythematosus.

Family history of AD or inflammatory disorder was even more common, with 76% of all relatives having had at least one AD or inflammatory disorder. The most frequent were thyroid disease (27%), rheumatoid arthritis/juvenile idiopathic arthritis (24%), and psoriasis (16%). In total, 76% of individuals in the cohort had a relative with at least one AD and/or inflammatory disorder (Table 4).

### *Post hoc* analyses

Given the high rates of AD in the cohort and their relatives, we conducted unplanned analyses comparing an autoimmune group (either the patient or a first-degree relative had at least one autoimmune disorder; *n* = 26) and a nonautoimmune group (neither the patient nor the first-degree relative had an autoimmune disorder; *n* = 19). No statistically significant differences emerged between these groups regarding any of the sociodemographic, clinical, or laboratory variables (Supplementary Tables S1, S2, S3, S4, S5).

## Discussion

PANS is a relatively new descriptive entity of unclear validity and still uncertain clinical utility. There is a great need for better characterization and long-term follow-up of this patient group to identify clinical and biological markers to guide treatment development. The PANS entity describes a wide range of symptoms, presenting a clinical challenge both when trying to assess symptoms and to evaluate treatments. In our experience, global functioning scales, such as CGI and CGAS, were easier to administer than syndrome-specific instruments such as the CYBOCS.

All patients included in the cohort fulfilled PANS criteria according to patient records, psychiatric and medical histories, and the clinical evaluation. In some cases, the time between symptom onset and presentation at the clinic/first assessment was several years (range 0.04–10.8) and the time of symptom onset was therefore more difficult to accurately assess. Compared with previously reported data from the Stanford cohort (Frankovich et al. 2015), the patients included in our cohort were ∼2 years younger at onset (7.5 vs. 9.6 years), but had almost the same delay before presenting at the clinic (presenting at 9.0 vs. 11.8 years). We observed a more even distribution between males and females (56% males in our cohort vs. 77% males in the Stanford cohort).

PANS is sometimes described as a disorder striking previously healthy children. This is not the clinical picture we observe in our cohort as almost a fifth of the patients (18%) had a previous psychiatric or neuropsychiatric diagnosis. In these patients, the PANS onset led to a sudden and severe worsening of psychiatric symptoms as well as development of a wider symptom spectrum. Interestingly, almost a quarter of the patients (24%) had a previous AD or inflammatory disorder and as many as 16% had an onset of an AD or an inflammatory disorder in temporal relation to the PANS onset.

We noted that somatic symptoms, such as enuresis, pain, and skin rashes, were less often reported if not asked or looked for specifically. Even though the somatic examination revealed relatively few abnormalities in most patients, it needs to be performed systematically to detect more severe somatic signs that would otherwise go unnoticed. This demonstrates the benefits of using a multispecialist team focusing on both psychiatric and medical issues.

An association of AD and ANAs with psychiatric states has previously been described (Graus et al. 2010; Najjar et al. 2012; Kayser and Dalmau 2011). Likewise, immunological phenotypes within both humoral and cellular immunity arms have been reported to be abnormal in individuals with psychiatric disease, and specific serum biological biomarkers have been determined for major depressive disorders (Zandi et al. 2011; Najjar et al. 2013). Furthermore, inflammatory markers such as CRP, IL-6, and TNF-α have been associated with development or severity of psychiatric disorders such as depression, psychosis, and bipolar disorder (Najjar et al. 2013; Goldstein et al. 2015; Khandaker et al. 2018a, 2018b).

We did not record any clear elevations in CRP, but we did not use the high-sensitivity CRP assay, which might be more accurate. In addition, we have only measured cytokine levels systematically in blood. For more complete results, these levels should be assessed in the CSF. In addition, as it is known that immune cell population activity varies according to circadian rhythms, it will be important in the future to include a defined time of the day for sampling of blood in case this contributed to some of the variation.

The fact that 37% of the sample had complement activation, 17% had a positive ANA value, 11% had anti-TPO antibodies, and 20% had immunoglobulin abnormalities, taken together with frequent indication of AD in our cohort, is suggestive of immune dysregulation in these individuals. Whether there is a causal relationship between these immunological markers and disease prognosis is currently unclear, but their value as biomarkers of PANS warrants further investigation.

The cohort needs to be expanded and recruitment of suitable control groups would be required (e.g., a regular OCD sample without suspected PANS and healthy age-matched controls) to establish whether these potentially interesting findings have clinical utility. However, we do know that the complement system is a key player in autoimmunity and activation rarely seen in healthy individuals (McGeer et al. 2017). In addition, anti-TPO antibodies are described to be prevalent in only 1%–2% healthy children (Harel et al. 2006; Kroon et al. 2013). Interpretation of ANAs is more difficult as the ANA test has a high false positive rate, meaning that many children who do not have an AD can have elevated ANA levels. Studies have indicated that up to 15% of healthy children may have a positive ANA test and that it may be frequent in children with nonautoimmune inflammatory diseases, including both acute and chronic infections (Wananukul et al., 2005; Malleson et al. 2010).

Previous epidemiological studies have suggested a familial link between AD and OCD and tic disorders, but it remains unclear if this link is stronger in patients also fulfilling criteria for PANS (Pérez-Vigil et al., 2016; Mataix-Cols et al. 2018). However, dividing the total cohort into an autoimmune group consisting of diagnosed AD in the patient or a first-degree relative and a nonautoimmune group consisting of no diagnosed AD in either the patient or a first-degree relative did not generate any significant differences. The high prevalence of AD or inflammatory disorders in the patients and their relatives has also been reported from the Stanford cohort (Frankovich et al., 2015) and may suggest a genetic vulnerability in families with AD to present with the constellation of symptoms described as PANS. However, while the presence and/or family history of AD may be a common feature in this patient group, more research is needed before this knowledge can guide diagnosis or treatment. The absence of AD in childhood does not necessarily mean that a person cannot fulfill the criteria for PANS as the onset of most ADs tends to occur later in life. Long-term follow-up of these patients using the Swedish health care registers will shed more light on the possible development of AD later in life.

It is important to note that many of the patients referred to our team with a suspected PANS-like presentation did not fulfill the regular PANS criteria or more stringent criteria that we used for inclusion in our cohort requiring the presence of somatic signs. The main reasons for exclusion from our cohort were absence of core symptoms such as OCD and/or anorexia, insidious symptom onset, and spontaneous resolution of symptoms before the assessment took place. However, many of these patients clearly exhibited an atypical onset of severe psychiatric symptoms, sometimes related to an infection or to an AD. While we did not include these patients in our cohort, they were clearly disabled by their symptoms. Whether the PANS criteria are too narrow and need to be broadened to include psychiatric manifestations other than OCD/anorexia triggered by autoimmune or inflammatory disorders is an important area of enquiry.

There is a great deal of misinformation among the general public and professionals alike regarding the nature of PANS, which may lead to inadequate or unnecessary treatments (Gilbert et al. 2018). During the course of our study, it became more apparent that patients were often treated with long-term antibiotics and/or NSAIDs and, in a few patients, even IVIG, without having had a systematic assessment of their suspected PANS symptoms. For this reason, our team started to routinely taper down current medications aimed at treating the suspected PANS symptoms when the patient first presented at our clinic. Fortunately, referrals have become progressively more accurate and most of the patients currently referred to our team do meet the criteria for PANS. These results highlight the importance of educating colleagues and the public about PANS since they appear to represent a small fraction of all cases referred to specialist OCD and related disorder clinics such as ours. During the study period, more than 1500 patients were referred to our specialist OCD and related disorders clinic and only 47 (or ∼3%) met criteria for PANS.

The main strength of the study is the careful protocol-driven approach of data collection and the multidisciplinary nature of our team. However, the study was conducted in a naturalistic clinical setting, which means that some data points differed across patients. For example, more chronic or severe patients had blood tests taken more regularly. For those patients who had tests taken on different occasions, we tried to capture an active disease state, but this was not easy to establish using objective criteria. For instance, some patients had positive laboratory results when they were in a less severe phase of their illness, and these results are therefore not considered. Serologies and cultures were only taken when considered clinically motivated, which may be considered as a limitation. Antistreptococcal antibody titers were not part of the protocol since they may be elevated in many preschool and school children who are frequently exposed to streptococci in everyday life and their validity is therefore disputed (Delice et al. 2015). In an attempt to select a strictly defined cohort and minimize risk of misdiagnosis, we did not include patients who did not exhibit any kind of somatic symptom or sign. Somatic signs are not part of main PANS criteria, but are listed as secondary diagnostic criteria. Therefore, our cohort may differ from other cohorts that did not have such a requirement. Additional limitations include parent-reported data on family history; the modest sample size, resulting in limited power for subgroup analyses or analyses of clinical trajectories; and relatively short follow-up time. However, we plan to follow these patients long term. As mentioned above, we cannot be sure if any of the potential biomarkers identified in this study are clinically useful until we compare results with those of a regular OCD and related disorders cohort. This work is currently ongoing.

## Conclusions

We have successfully established a multidisciplinary PANS team and an assessment protocol. There was a strong indication of an association with AD in our cohort. Further work is needed to establish whether any of the potential biomarkers identified will be clinically useful. Long-term follow-up of these patients using the Swedish national registers will enable a deeper understanding of the course of this patient group.

## Clinical Significance

A thorough multidisciplinary assessment is needed to confirm that patients fulfill strict criteria for PANS or PANS-like presentations, rather than regular OCD or other psychiatric disorders, as this will determine whether antibiotic, anti-inflammatory, or immunomodulatory treatments should be considered. The high rates of autoimmunity in this patient group and their families warrant close collaboration between psychiatric and pediatric services.

## Supplementary Material

Supplemental data

Supplemental data

Supplemental data

Supplemental data

Supplemental data
