# Supplementary material for: Establishing a Pediatric Acute-Onset Neuropsychiatric Syndrome Clinic: Baseline Clinical Features of the Pediatric Acute-Onset Neuropsychiatric Syndrome Cohort at Karolinska Institutet
Source: J Child Adolesc Psychopharmacol. 2019 Oct 7;29(8):625–33. doi: 10.1089/cap.2018.0127 (PMC6786340; doi:10.1089/cap.2018.0127)
Supplement: Supplemental data [file Supp_Table1.pdf]

Supplementary Data

SUPPLEMENTARY TABLE S1. COMPARISON OF PATIENT CHARACTERISTICS DIFFERENTIATING AUTOIMMUNE DISEASE AND NONAUTOIMMUNE DISEASE GROUPS AT INTAKE ($N=45$)

Patient demographics	*Total cohort (n = 45)*	*AD (n = 26)*	*Non-AD (n = 19)*	*Chi-square/ t-test (df)*	p-*Value*
Male	25/45 (56%)	15/26 (58%)	10/19 (53%)	0.11	0.736
Mean age at symptom onset (years)	7.5 (SD 2.5)	7.4 (SD 2.7)	7.6 (SD 2.2)	0.37 (42.5)	0.710
Mean age at intake (years)	9.0 (SD 3.1)	9.1 (SD 3.5)	8.9 (SD 2.7)	–0.21 (42.7)	0.833
Developmental abnormalities (psychomotor, language disorder, and/or learning disability)	8/45 (18%)	2/26 (8%)	6/19 (32%)	0.04	0.053
Preexisting psychiatric/neuropsychiatric diagnoses	8/45 (18%)	4/26 (15%)	4/19 (21%)	0.24	0.624
Preexisting autoimmune disease or inflammatory disorder	11/45 (24%)	11/26 (42%)	0	—	—
CGAS at intake[a]	50 (SD 10.1) (*n* = 43)	50 (SD 11.6) (*n* = 24)	49 (SD 8.3) (*n* = 19)	–0.32 (40.7)	0.752
CGI-S at intake	3.8 (SD 0.9)	3.8 (SD 1.0)	3.8 (SD 0.9)	–0.20 (42.0)	0.840
Acute onset	42/45 (93%)	25/26 (96%)	17/19 (89%)	0.78	0.394
Infection in temporal relation to symptom onset	42/45 (93%)	25/26 (96%)	17/19 (89%)	0.78	0.394
Onset of autoimmune disease or inflammatory disorder in temporal relation to symptom onset	7/45 (16%)	7/26 (27%)	0	—	—

[a]Available for 43 patients only.

AD, autoimmune disease; AD group, diagnosis of AD in the patient or first-degree relative; CGAS, Children's Global Assessment Scale; CGI-S, Clinical Global Impressions-Severity Scale; non-AD group, neither the patient nor first-degree relative has a diagnosis of AD; SD, standard deviation.
