# Supplementary material for: Establishing a Pediatric Acute-Onset Neuropsychiatric Syndrome Clinic: Baseline Clinical Features of the Pediatric Acute-Onset Neuropsychiatric Syndrome Cohort at Karolinska Institutet
Source: J Child Adolesc Psychopharmacol. 2019 Oct 7;29(8):625–33. doi: 10.1089/cap.2018.0127 (PMC6786340; doi:10.1089/cap.2018.0127)
Supplement: Supplemental data [file Supp_Table2.pdf]

Supplementary Table S2. Onset Symptoms as Described in Patient Records and/or at Presentation at the Clinic, Differentiating Autoimmune Disease and Nonautoimmune Disease Groups

Symptom onset/presentation	*Total cohort* (n = *45)*	*AD* (n = *26)*	*Non-AD* (n = *19)*	*Chi-square*	*Significance* (p*-value)*
Obsessive-compulsive disorder	40/45 (89%)	24/26 (92%)	16/19 (84%)	0.72	0.402
Eating disorder (anorexia/avoidant restrictive food intake disorder)	18/45 (40%)	10/26 (38%)	8/19 (42%)	0.06	0.805
Anxiety (including separation anxiety)	35/45 (78%)	20/26 (77%)	15/19 (79%)	0.75	0.872
Emotional lability/depression	32/45 (71%)	18/26 (69%)	14/19 (74%)	0.11	0.745
Irritability/aggression	20/45 (44%)	13/26 (50%)	7/19 (37%)	0.77	0.382
Regression	18/45 (40%)	9/26 (35%)	9/19 (47%)	0.74	0.390
Tics	26/42 (62%) (*n* = 42)	12/25 (48%) (*n* = 25)	14/17 (82%) (*n* = 17)	5.36	0.031
Motor abnormalities	27/45 (60%)	15/26 (58%)	12/19 (63%)	0.14	0.712
Sensor abnormalities	18/36 (50%) (*n* = 36)	11/21 (52%) (*n* = 21)	7/15 (47%) (*n* = 15)	0.11	0.735
Sleeping disorder	31/45 (69%)	20/26 (77%)	11/19 (58%)	1.84	0.178
Hyperactivity	18/42 (43%) (*n* = 42)	9/24 (38%) (*n* = 24)	9/18 (50%) (*n* = 18)	0.66	0.419
Attention deficit	22/35 (63%) (*n* = 35)	10/19 (53%) (*n* = 19)	12/16 (75%) (*n* = 16)	1.90	0.178
Deterioration in school performance	17/34 (50%) (*n* = 34)	11/19 (58%) (*n* = 19)	6/15 (40%) (*n* = 15)	1.08	0.303
Urinary problems (enuresis)	18/41 (44%) (*n* = 41)	9/24 (38%) (*n* = 24)	9/17 (53%) (*n* = 17)	0.96	0.329
Pain	14/37 (38%) (*n* = 37)	8/22 (36%) (*n* = 22)	6/15 (40%) (*n* = 15)	0.05	0.823

AD, autoimmune disease; AD group, diagnosis of AD in the patient or first-degree relative; non-AD group, neither the patient nor first-degree relative has a diagnosis of AD.
