# Supplementary material for: Establishing a Pediatric Acute-Onset Neuropsychiatric Syndrome Clinic: Baseline Clinical Features of the Pediatric Acute-Onset Neuropsychiatric Syndrome Cohort at Karolinska Institutet
Source: J Child Adolesc Psychopharmacol. 2019 Oct 7;29(8):625–33. doi: 10.1089/cap.2018.0127 (PMC6786340; doi:10.1089/cap.2018.0127)
Supplement: Supplemental data [file Supp_Table3.pdf]

Supplementary Table S3. Somatic Findings at First Clinical Evaluation at the Clinic, Differentiating Autoimmune Disease and Nonautoimmune Disease Groups

Somatic sign onset/presentation	*Total cohort* (n=*45)*	*AD* (n=*26)*	*Non-AD* (n=*19)*	*Chi-square*	*Significance* (p-*value)*
Abnormalities in somatic assessment of:					
Heart	1/28 (4%) (*n*=28)	0/17 (0%) (*n*=17)	1/11 (9%) (*n*=10)	—	—
Lungs	0/27 (0%) (*n*=27)	0/18 (0%) (*n*=18)	0/9 (0%) (*n*=9)	—	—
Stomach	0/13 (0%) (*n*=13)	0/7 (0%) (*n*=7)	0/6 (0%) (*n*=6)	—	—
Joints	0/16 (0%) (*n*=16)	0/11 (0%) (*n*=11)	0/5 (0%) (*n*=5)	—	—
Ear, nose, and throat infection signs	12/26 (46%) (*n*=26)	8/17 (47%) (*n*=17)	4/9 (44%) (*n*=9)	0.02	0.899
Palatal petechiae	2/22 (10%) (*n*=22)	1/14 (7%) (*n*=14)	1/8 (13%) (*n*=8)	0.17	0.678
Skin abnormalities (dermatographia, livedo, severe dryness, redness, rash, or eczema)	11/18 (61%) (*n*=18)	6/11 (55%) (*n*=11)	5/7 (71%) (*n*=7)	0.52	0.477
Neurological abnormalities	7/31 (23%) (*n*=31)	3/20 (15%) (*n*=20)	4/11 (36%) (*n*=11)	1.79	0.185
Abnormalities in motor function	7/31 (23%) (*n*=31)	4/19 (21%) (*n*=19)	3/11 (27%) (*n*=11)	0.15	0.699
Chorea	1/41 (2%) (*n*=41)	1/24 (4%) (*n*=24)	0/17 (0%) (*n*=17)	—	—
Choreiform movements	6/26 (23%) (*n*=26)	3/17 (18%) (*n*=17)	3/9 (33%) (*n*=9)	0.79	0.373

AD, autoimmune disease; AD group, diagnosis of AD in the patient or first-degree relative; non-AD group, neither the patient nor first-degree relative has a diagnosis of AD.
