# Supplementary material for: Establishing a Pediatric Acute-Onset Neuropsychiatric Syndrome Clinic: Baseline Clinical Features of the Pediatric Acute-Onset Neuropsychiatric Syndrome Cohort at Karolinska Institutet
Source: J Child Adolesc Psychopharmacol. 2019 Oct 7;29(8):625–33. doi: 10.1089/cap.2018.0127 (PMC6786340; doi:10.1089/cap.2018.0127)
Supplement: Supplemental data [file Supp_Table4.pdf]

Supplementary Table S4. Laboratory Findings Differentiating Autoimmune Disease and Nonautoimmune Disease Groups

Laboratory findings	*Total cohort (*n=*45)*	*AD (*n=*26)*	*Non-AD (*n=*19)*	*Chi-square*	*Significance (*p*-value)*
CBC abnormalities	26/45 (58%)	15/26 (58%)	11/19 (58%)	0	0.989
Elevated ESR	6/43 (14%) (*n*=43)	3/26 (12%)	3/17 (18%) (*n*=17)	0.31	0.574
Elevated CRP	4/37 (11%) (*n*=37)	1/21 (5%) (*n*=21)	3/16 (19%) (*n*=19)	1.86	0.206
Low ferritin	8/39 (21%) (*n*=39)	4/24 (17%) (*n*=24)	4/15 (27%) (*n*=15)	0.56	0.455
Low vitamin D	10/36 (28%) (*n*=36)	6/21 (29%) (*n*=21)	4/15 (27%) (*n*=15)	0.02	0.900
TSH abnormalities	4/40 (10%) (*n*=40)	3/25 (12%) (*n*=25)	1/15 (7%) (*n*=15)	0.31	0.591
Low T4	0/40 (0%) (*n*=40)	0/25 (0%) (*n*=25)	0/15 (0%) (*n*=15)	—	—
Anti-TPO	4/38 (11%) (*n*=38)	3/25 (12%) (*n*=25)	1/13 (8%) (*n*=13)	0.18	0.684
Transglutaminase antibodies	2/40 (5%) (*n*=40)	2/25 (8%) (*n*=25)	0/15 (0%) (*n*=15)	—	—
ANA	7/41 (17%) (*n*=41)	5/25 (20%) (*n*=25)	2/16 (13%) (*n*=16)	0.40	0.537
Histone antibodies	0/31 (0%) (*n*=31)	0/21 (0%) (*n*=21)	0/10 (0%) (*n*=10)	—	—
Elevated SAA	3/22 (14%) (*n*=22)	1/14 (7%) (*n*=14)	2/8 (25%) (*n*=8)	1.32	0.267
Complement activation	13/35 (37%) (*n*=35)	7/22 (32%) (*n*=22)	6/13 (46%) (*n*=13)	0.71	0.399
Low IgG	4/36 (11%) (*n*=36)	2/22 (9%) (*n*=22)	2/14 (14%) (*n*=14)	0.23	0.631
Low IgA	3/34 (9%) (*n*=34)	3/22 (14%) (*n*=22)	0/12 (0%) (*n*=12)	—	—
Low IgM	0/34 (0%) (*n*=34)	0/22 (0%) (*n*=22)	0/12 (0%) (*n*=12)	—	—
Elevated IL-1-β	0/25 (0%) (*n*=25)	0/14 (0%) (*n*=14)	0/9 (0%) (*n*=9)	—	—
Elevated IL-6	1/25 (4%) (*n*=25)	0/15 (0%) (*n*=15)	1/11 (9%) (*n*=11)	—	—
Elevated IL-8	0/25 (0%) (*n*=25)	0/14 (0%) (*n*=14)	0/11 (0%) (*n*=11)	—	—
Elevated TNF-α	0/24 (0%) (*n*=24)	0/14 (0%) (*n*=14)	0/10 (0%) (*n*=10)	—	—
Positive strep/throat culture	5/10 (50%) (*n*=10)	4/8 (50%) (*n*=8)	1/2 (50%) (*n*=2)	0	1.000

AD, autoimmune disease; AD group, diagnosis of AD in the patient or first-degree relative; ANA, antinuclear antibodies; CBC, complete blood count; CRP, C-reactive protein; ESR, erythrocyte sedimentation rate; IgA, immunoglobulin A; IgM, immunoglobulin M; IL, interleukin; non-AD group, neither the patient nor first-degree relative has a diagnosis of AD; SAA, serum amyloid A; TNF-α, tumor necrosis factor-α; TPO, thyroperoxidase; TSH, thyroid-stimulating hormone.
