# Supplementary material for: Establishing a Pediatric Acute-Onset Neuropsychiatric Syndrome Clinic: Baseline Clinical Features of the Pediatric Acute-Onset Neuropsychiatric Syndrome Cohort at Karolinska Institutet
Source: J Child Adolesc Psychopharmacol. 2019 Oct 7;29(8):625–33. doi: 10.1089/cap.2018.0127 (PMC6786340; doi:10.1089/cap.2018.0127)
Supplement: Supplemental data [file Supp_Table5.pdf]

Supplementary Table S5. Psychiatric Disorders in First-, Second-, and Third-Degree Relatives, Differentiating Autoimmune Disease and Nonautoimmune Disease Groups

Family history	*Total cohort* (n=*45*)	*AD* (n=*26*)	*Non-AD* (n=*19*)	*Chi-square*	*Significance* (p-*value*)
Psychiatric/neuropsychiatric disorder in first-, second-, and third-degree relatives					
Attention-deficit/hyperactivity disorder	9/45 (20%)	4/26 (15%)	5/19 (26%)	0.81	0.370
Autism spectrum disorder	5/45 (11%)	3/26 (12%)	2/19 (11%)	0.01	0.915
Tics	6/45 (13%)	5/26 (19%)	1/19 (5%)	2.05	0.202
Obsessive-compulsive disorder	7/45 (16%)	4/26 (15%)	3/19 (16%)	0	0.970
Anxiety disorder	7/45 (16%)	3/26 (12%)	4/19 (21%)	0.75	0.390
Depression	16/45 (36%)	7/26 (27%)	9/19 (47%)	2.00	0.161
Any	29/45 (64%)	16/26 (62%)	13/19 (68%)	0.23	0.634

AD, autoimmune disease; AD group, diagnosis of AD in the patient or first-degree relative; non-AD group, neither the patient nor first-degree relative has a diagnosis of AD.
